# Stress-induced mutation via DNA breaks in *Escherichia coli*: A molecular mechanism with implications for evolution and medicine

**DOI:** 10.1002/bies.201200050

**Published:** 2012-08-22

**Authors:** Susan M Rosenberg, Chandan Shee, Ryan L Frisch, P J Hastings

**Affiliations:** 1 Department of Molecular and Human Genetics, Baylor College of MedicineHouston, TX, USA; 2 Department of Biochemistry and Molecular Biology, Baylor College of MedicineHouston, TX, USA; 3 Department of Molecular Virology and Microbiology and the Dan L. Duncan Cancer Center, Baylor College of MedicineHouston, TX, USA

**Keywords:** DinB, DNA repair, SOS response, spontaneous mutation, stress response

## Abstract

Evolutionary theory assumed that mutations occur constantly, gradually, and randomly over time. This formulation from the “modern synthesis” of the 1930s was embraced decades before molecular understanding of genes or mutations. Since then, our labs and others have elucidated mutation mechanisms activated by stress responses. Stress-induced mutation mechanisms produce mutations, potentially accelerating evolution, specifically when cells are maladapted to their environment, that is, when they are stressed. The mechanisms of stress-induced mutation that are being revealed experimentally in laboratory settings provide compelling models for mutagenesis that propels pathogen–host adaptation, antibiotic resistance, cancer progression and resistance, and perhaps much of evolution generally. We discuss double-strand-break-dependent stress-induced mutation in *Escherichia coli.* Recent results illustrate how a stress response activates mutagenesis and demonstrate this mechanism's generality and importance to spontaneous mutation. New data also suggest a possible harmony between previous, apparently opposed, models for the molecular mechanism. They additionally strengthen the case for anti-evolvability therapeutics for infectious disease and cancer.

## Introduction

Mutations that drive evolution were assumed to form randomly, constantly, and gradually, independently of selective environments 1. This basic assumption has been challenged by the discoveries of mutation mechanisms in bacterial, yeast, and human cells that are activated during stress, controlled by stress response processes 2–4. Stress-inducible mutation mechanisms produce mutations, potentially increasing genetic diversity and the ability to evolve 5, specifically when cells are maladapted to their environment, that is, when they are stressed. These mechanisms could fuel the evolutionary arms races between pathogens and hosts, pathogens and chemotherapies, cancers and hosts, and cancers and chemotherapies; hence they are important to understand. Here we focus on a molecular mechanism of stress-induced mutation in *Escherichia coli*: double-strand break-dependent stress-induced mutation. In this mechanism, repair of DNA breaks by homologous recombination is switched from a high-fidelity (non-mutagenic) process to a mutagenic mode by activation of the RpoS general stress response. This stress response allows error-prone DNA polymerases to participate in repair specifically during stress, causing mutations and potentially accelerating evolution during stress. Recent work illustrates how a stress response can activate mutagenesis. Several long-stranding issues in this field are resolved by new work reviewed here. Excitingly, new demonstrations of the general importance of stress-induced mutagenesis strengthen the case for a new class of chemotherapies that would combat infectious diseases and cancers by inhibiting their ability to out-evolve both our immune system and current drugs.

Some long-standing tensions in the field have concerned the generality and the precise molecular mechanism of stress-induced mutation during DNA break repair. From 1995 6–8 to more recently 9, 10 DNA break-dependent stress-induced mutation was suggested to be peculiar to conjugative plasmids, and thus potentially not generally important. Various lines of recent work resolve this point definitively, showing that this mutation mechanism occurs in chromosomes of plasmid-free cells (11 and others reviewed below). Similarly, two models for the molecular mechanism of mutagenesis have competed: one in which mutations result from gene amplifications increasing the number of copies of genes that are mutated 9 and another in which mutations are associated with any act of DNA break repair that occurs when the RpoS response is activated (e.g. 12). New data suggest a possible harmony between these previous, apparently opposed models for mutation in stressed cells, and suggest that both might apply, at least in some circumstances. A very old conceptual problem is, in general, how could stress-induced mutation mechanisms have evolved? Could they be selected as evolutionary engines, or are they necessarily negative or neutral, but unavoidable, consequences of other aspects of a cell's biology? From 1975 13 to the present (e.g. 14–17), this question appeared intractable for mutagenesis associated with the SOS DNA-damage response. New understanding of the DNA break-dependent stress-induced mutation mechanism is addressing a key part of this long standing dilemma.

In this review we consider the impact of recent results on the temporal regulation of mutagenesis by stress responses. Two other non-random aspects of mutagenesis that may accelerate evolution via double-strand-break-dependent and other mutation pathways are reviewed elsewhere: potential localization of mutagenesis in genomic space (reviewed in 2, 12, 18), which could facilitate concerted evolution within genes and localized gene clusters; and restriction of increased mutation rates (or “hypermutation”) to small differentiated cell subpopulations 2, 19–21, which may mitigate risks to the whole population.

## Molecular mechanism: DSB-dependent SIM requires three simultaneous events

**Figure 1:**
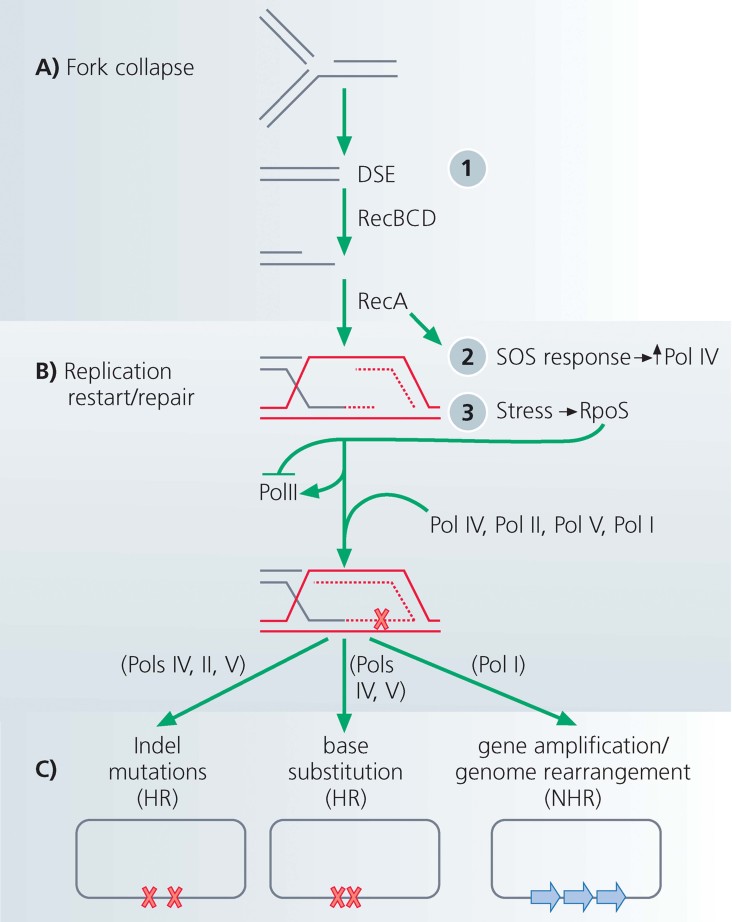
Three events are required for double-strand-break-dependent stress-induced point mutagenesis: (1) a DSB or double-strand end (DSE) and its repair; (2) activation of the SOS response, which upregulates PolIV/DinB error-prone DNA polymerase; and (3) a second stress that activates RpoS. RpoS allows Pols IV 11, 12, II 25, V 11, 31, and/or I 32, 33 to participate in break repair, instead of/in addition to high-fidelity DNA PolIII. We hypothesize that RpoS may license the use of these alternative DNA polymerases by down-regulating their competitor, DNA PolIII 25. **A**: Creation of DSE by replication-fork collapse. DSBs with two DSEs might also form spontaneously (not shown). Lines, single DNA strands. DSE repair in *E. coli* 91, 92 begins with digestion of the DSE by RecBCD enzyme. RecBCD produces single-strand (ss)DNA, then loads RecA recombinase onto it 93. **B**: The RecA-ssDNA filament searches for and finds an identical DNA sequence (red lines) to use as a template for repair synthesis (dashed red lines; e.g. a sister DNA molecule). RecBCD-mediated DSE repair uses the high-fidelity major replicative DNA polymerase PolIII 37 and is not mutagenic in unstressed growing cells 11, 12. Xs, DNA polymerase errors that become mutations. **C**: Mutated chromosomes. Single lines, double-stranded DNA; HR, homologous recombination; NHR, nonhomologous or microhomologous recombination; indel, 1-few bp insertion or deletion.

Double-strand-break-dependent stress-induced mutagenesis in *E. coli* occurs when three events occur simultaneously (Fig. 1) 11, 12: (i) the formation and repair by homologous recombination of a DNA double-strand break or double-strand end (DSB/DSE); (ii) induction of the bacterial DNA-damage response, the SOS response, which DSBs/DSEs induce 22, 23; and (iii) a second stress, unrelated to the DSB/DSE, that activates the general or starvation stress response controlled by the RpoS (*σ*^S^) transcriptional activator. These events occur and promote mutagenesis as follows.

DSBs/DSEs occur spontaneously in just over 10^−3^ of growing *E. coli* cells 23 and induce the SOS response about 25% of the time that they are repaired 23. Thus, event number 2, the SOS response, is a consequence of event 1, formation of a DSB (Fig. 1A and B).

We showed that the normally high-fidelity (non-mutagenic) process of repair of DSBs/DSEs by homologous recombination (HR) is switched to a mutagenic mode, using error-prone DNA polymerases (Pols) IV, II, and V, specifically when cells are stressed, under the control of the RpoS response 11, 12. The mutagenic repair causes −1 bp deletion (“indel”) and base-substitution mutations (Fig. 1). The demonstration of an RpoS-controlled switch to mutagenic break repair was made using technology for creating single DSBs at any site in the *E. coli* genome. First, we created a regulatable site-specific double-strand endonuclease for *E. coli* (I-*Sce*I, housed in the *E. coli* chromosome 24). Then we used it to engineer cells with inducible site-specific DSBs at locations of our choice, and assay mutation at reporter genes nearby, as follows.

We engineered the chromosomally encoded regulatable I-*Sce*I endonuclease, and a single I-*Sce*I cutsite, into *E. coli.* The cutsites caused DSBs 12, 24 in an F′ conjugative plasmid carrying a *lac* mutation-reporter gene 12, 25 or in the chromosome of plasmid-free cells 11, 23 carrying a *tet* mutation-reporter gene. When I-*Sce*I endonuclease was synthesized in starvation-stressed cells, the resulting DSBs cause a 50–6,000-fold increase in mutations at genes in the DNA molecule with the I-*Sce*I cutsite 11, 12, 25. The increased mutagenesis occurs only when both I-*Sce*I enzyme and cutsite are present (DSB-dependently), and only during starvation stress, or if the RpoS starvation–stress response is induced artificially in unstressed cells. When the I-*Sce*I cut was in an F′ plasmid with a *lac-*frameshift reporter gene nearby, the DSBs stimulated *lac* reversion mutagenesis ∼6,000-fold 12. By contrast, I-*Sce*I cuts made in a different plasmid, in trans to *lac*, increased *lac* reversion in the F′ only threefold 12. The DSB made in trans to *lac* could, however, activate *lac* reversion if the DNA next to *lac* contained sequences identical to one end of the trans-cut plasmid. That is, a DSE in another molecule provoked mutation at *lac* if that DSE could interact by homologous recombination with the DNA near *lac*. This demonstrates that DSE repair by homologous recombination causes the mutations, and the mutations happen in DNA molecules that are engaged in repair 12.

In starved plasmid-free cells, chromosomal I-*Sce*I cuts provoke reversion of a *tet* +1 bp frameshift allele, which confers resistance to the antibiotic tetracycline 11. The mutagenesis requires DSB-repair proteins RecA, RecBC and RuvABC, PolIV error-prone DNA polymerase, the SOS response, which upregulates PolIV, and the RpoS stress response 11, similarly to DSB-dependent stress-induced mutation in the F′-based Lac assay 12. The DSB-dependent Tet-resistant mutants were shown to be produced dependently on the time spent in starvation (prolonged stationary phase). Unlike the F′ Lac assay, the starvation stress applied did not impose any selection for the function of the chromosomal *tet* gene, which was assayed after release from starvation. This distinction is important for models of mutagenesis considered below (Solutions to old problems and possible harmony).

## The RpoS stress response throws the switch to mutagenic break repair

DSEs induce the SOS DNA-damage response 22, 23, which upregulates ∼40 DNA-damage-inducible genes transcriptionally. Of them only *dinB*, encoding PolIV, is required at SOS-induced levels for DSE-dependent stress-induced mutagenesis 26. However, even with PolIV upregulated 10-fold, DSE repair is not mutagenic and does not appear to use PolIV unless the RpoS response is also induced 11, 12, 27, 28. That is, the cell must sense a second stress, in addition to DNA damage, for mutagenesis to ensue. The RpoS response is activated by starvation, osmotic, cold, pH, and oxidative stresses 29: it both down-regulates and transcriptionally up-regulates hundreds of genes that protect cells from stress. (Pol IV also aids replication-fork repair during massive replication failures, but whether this was RpoS-dependent was not tested 30.)

The RpoS response throws a switch that allows error-prone DNA polymerases to be used in DSE repair and thus limits mutagenesis to times of RpoS-inducing stress 11, 12. RpoS upregulates PolIV twofold 27, and somehow licenses the use of PolsIV 11, 12, II 25, V 11, 31, and I 32, 33 in DSE repair, all of which cause mutations 25 (Fig. 1B and C). This might involve the transcription factor NusA, which binds PolIV 34 and is required for mutagenesis 35. RpoS throws this switch either during stress, or if RpoS is upregulated artificially in unstressed cells 11, 12. That is, stress itself is not necessary; activation of the RpoS response in unstressed cells that have an I-*Sce*I-produced DSB was sufficient to cause mutations during repair of the DSB 11, 12.

RpoS appears to allow the use of four lower-fidelity DNA polymerases in DSE/DSB repair, creating various kinds of mutations. PolIV promotes 85% 36 and PolII the remaining 15% 25 of −1 bp frameshift mutations, PolV contributing slightly to the PolIV-dependent component 11. PolsIV and V also promote DSE- and RpoS-dependent base substitutions 11, 31. PolI, however, is required for DSE- 12, 33 and RpoS-dependent 28 stress-induced genomic rearrangements including gene amplifications 32, 33 (Fig. 1C). The use of these four DNA polymerases during RpoS-inducing stress contrasts with DSE repair in unstressed cells, which requires the high-fidelity major replicative DNA polymerase, PolIII 37, and is non-mutagenic 11, 12.

The mechanism by which RpoS allows error-prone DNA pols to participate in repair is unknown. We suggested that RpoS might promote mutagenic DSB/DSE repair by inhibiting PolIII so that the other four *E. coli* DNA polymerases have access to the DSB repair replisome 25 (Fig. 1). This hypothesis remains to be tested. All of the DNA pols appear to compete 38–42 and if cells are not stressed, or do not have RpoS activated, PolIII appears to win, and repair is non-mutagenic. Thus, DSE-dependent stress-induced mutagenesis is controlled critically by RpoS, which switches DSE repair from a high-fidelity mode using PolIII to an error-prone mutagenic process, using error-prone DNA polymerases when cells are stressed, potentially accelerating evolution specifically under stress.

## An old idea revived: Could mutagenesis be a selected trait?

In the context of the SOS DNA-damage response, Radman 13, then Echols 22, realized that increased mutagenesis during stress could accelerate evolution and suggested that SOS mutagenesis might have been selected for its evolution-promoting ability. However, from its original proposal in 1975 6 to present (e.g. 8–11), mutagenesis associated with the SOS response has been argued to be an unavoidable consequence of the need to repair DNA, not a property selected in its own right for promoting the ability to evolve. These opposed views cannot be resolved in the context of the SOS response because SOS *is* required for repair and survival of DNA damage 43. By contrast, in DSB-dependent stress-induced mutation, neither RpoS nor PolIV enhances survival or repair of DSBs during stress, which works as efficiently 11 or more efficiently 12 without them. That is, the cell *did* evolve efficient non-mutagenic break repair, which it uses if RpoS or PolIV are knocked out. Therefore, though both RpoS and PolIV contribute to survival of problems other than DSEs 29, 44, their roles in mutation during DSE repair are demonstrably not an unavoidable consequence of the need to repair DSEs. Thus, it is possible that RpoS- and PolIV-promoted mutagenic break repair might have evolved based on selection for its properties as an evolutionary engine.

## Spontaneous mutations occur by DNA break-dependent stress-induced mutagenesis

The experiments demonstrating the RpoS-controlled switch to mutagenic break repair in the *E. coli* chromosome 11 and an F′ plasmid 12 examined mutagenesis at artificially created DSBs. Importantly, when no I-*Sce*I is used, half of the *spontaneous* frameshift and base-substitution mutagenesis in the chromosomes of starved plasmid-free *E. coli* also requires the same PolIV, SOS, RpoS, and DSB-repair proteins 11. Even the highly DSE-specific RecBCD enzyme is required 11. The data imply that spontaneous DSBs/DSEs instigate stress-induced mutagenesis in the chromosome normally, and do so similarly as at I-*Sce*I cuts 11. Spontaneous mutagenesis has long been an intractable problem because many pathways contribute, making identification of the proteins required or mechanism for any of them difficult 45. These findings demonstrate that DSB-dependent stress-induced mutation is an important source of spontaneous mutations in *E. coli*, and solve a substantial piece of the problem of how spontaneous mutations arise.

## Solutions to old problems and possible harmony

Recent results help resolve some old problems and suggest harmony between previous differing views of mutagenesis in stressed bacteria. Before I-*Sce*I, mechanisms of DSB-dependent stress-induced mutation and amplification 46, 47 (not discussed here, see 48, 49) had been studied mostly using an F′ conjugative plasmid-borne Lac-reversion assay 50. In the “classical” Lac assay, spontaneous DSEs instigated in F by its transfer endonuclease 12 cause very high level DSB-repair-protein- 51–53, SOS- 26, 50, 54, RpoS- 27, 28, and PolIV- 36 dependent mutation. A concern about interpreting results from the Lac assay was that the mutation mechanism might be specific to the F′ 9. Now that the same mechanism has been demonstrated in the chromosomes of plasmid-free cells, both at I-*Sce*I-induced DNA breaks and spontaneously 11, this concern can be put to rest.

A second concern was that the stress that resulted in mutagenesis (presumably starvation) might merely have selected the Lac^+^ mutants, rather than induced their formation. In one model 9, a preexisting *lac* gene duplication might undergo amplification. Under selection on lactose medium for the increased production of beta-galactosidase from the weakly-function *lac* gene, the amplification might allow extensive DNA replication. Mutations might then occur independently of stress in one of the many replicated copies 9. This conceptual problem, of whether stress induces or merely selects mutations, was resolved by measuring DSB-dependent stress-induced mutagenesis under starvation stress conditions that do not select the mutation assayed. Specifically, the Tet assay was used: cells with a *tet* frameshift mutation are starved in the absence of the antibiotic tetracycline, then rescued from starvation and Tet-resistant mutants quantified 11, 12. The mutagenesis depended on the time that cells were left starving 11. Neither *tet* function nor the functions of any nearby genes either in the F′ 12 or chromosome 11, were selected during this time. The Tet assay allowed the clean conclusion that mutagenesis was induced not selected by starvation stress. This conclusion was also suggested by two earlier chromosomal assays of RecB (DSB)-, PolIV- 31, 55, and RpoS-dependent 31 *tet* and *amp* gene mutations during starvation in the absence of selection for the antibiotic resistance 31, 55. The model in which stress selects rather than induces mutations was also addressed by showing that the RpoS-dependent switch to error-prone break repair occurred independently of stress (selection), when RpoS was expressed in unstressed cells 11, 12. It is now clear that the RpoS response induces mutagenesis independently of selection for any particular mutation (shown also by 94).

**Figure 2:**
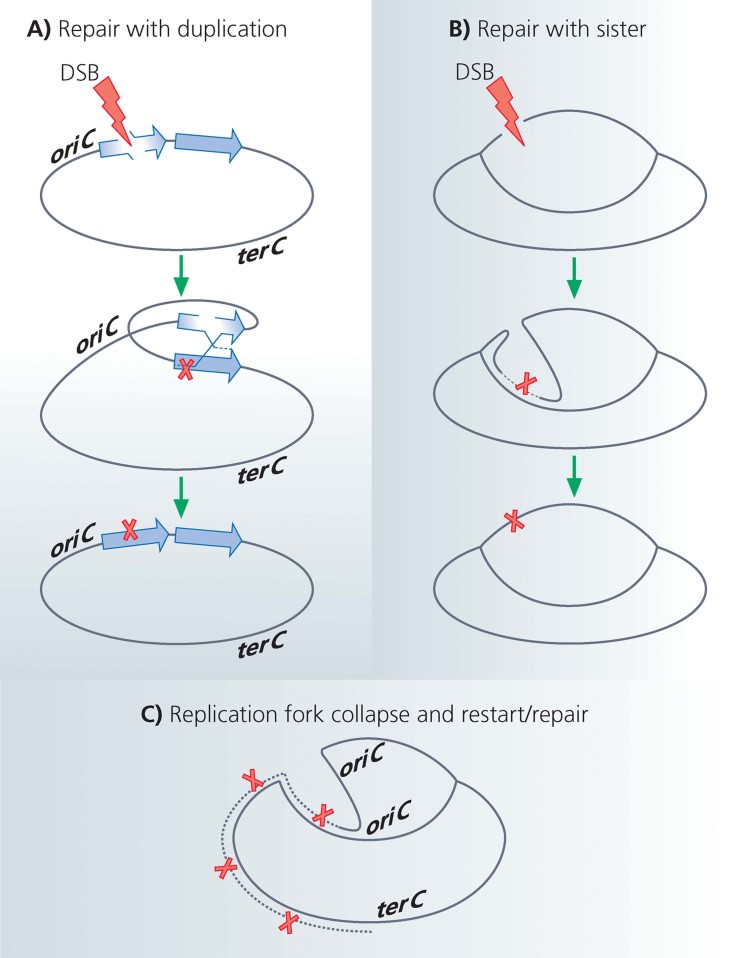
Repair of DSBs during growth-limiting stress could be via homologous interaction with a sister DNA molecule or a duplicated DNA segment. **A**: Repair of a two-ended double-strand break (DSB) might sometimes or often require an identical duplicated DNA segment in the chromosomes of those starved cells in which there is no sister chromosome for repair. **B**: Repair of a DSB with a sister molecule. This route might be common even during starvation in F′ conjugative plasmids, because they are higher copy than the bacterial chromosome. **C**: Repair of a single double-strand end (DSE) caused by replication fork collapse and restart would be expected to use a sister DNA molecule because forks collapse during replication, when there is a sister. However, whether all or most spontaneous DSEs result from fork collapse is not known 23. Other mechanisms of creation of spontaneous DSEs are possible. Single lines, double-strand DNA; blue arrows, duplicated DNA segments; dashed lines, newly synthesized DNA; red Xs, DNA polymerase errors that become mutations.

However, the idea of gene duplications as a precursor to mutagenesis in stressed cells is attractive, and both kinds of models might be right, as suggested by new results 56. We suggested that gene duplications might be the usual source of DNA sequence homology with which HR repairs the breaks that produce chromosomal stress-induced mutations 56. This could help explain the observation that DSB-dependent mutant frequency is lower in the chromosome than the F′, even when other *trans*- and *cis*- acting variables are controlled between these two molecules 56. A simple explanation is that the F is higher copy (2–3 for every chromosome), and so may usually have a sister molecule with which to repair (Fig. 2B and C), whereas during starvation the chromosome may be limited to duplications as repair partners (Fig. 2A). Although 40% of stationary *E. coli* are reported to carry two chromosomes 57, and only ∼10^−3^ of *Salmonella* to carry a spontaneous duplication 9, the source of the repair partner in starving haploid bacteria has not been determined, and the duplication model might predominate under the specific starvation conditions that promote DSB-dependent stress-induced mutation, particularly in the chromosome.

## RpoS- and/or DSB-dependent stress-induced mutation mechanisms seen in other circumstances

Error-prone DSB repair appears to underlie stress-induced mutagenesis in circumstances other than starvation and in organisms other than *E. coli* (reviewed in 2). *E. coli* under antibiotic stress induce a similar DSB-repair-protein-, SOS-, and PolIV/II/V-dependent mutagenesis pathway 58, 59. *Pseudomonas aeruginosa* biofilms show DSB- and DSB-repair-protein-dependent generation of genetic diversity which may arise by a similar mechanism 60. Pathogenic *Salmonella* induce DSB-repair-protein-, SOS-, PolIV-, and RpoS-dependent mutation in response to bile, a membrane irritant (61 and J. Casadesus, personal communication). Although RpoS- and PolIV-dependent mutagenesis was not seen in non-pathogenic Salmonella strain LT2 62, 63, this probably reflects the fact that LT2 is a natural variant that is nonpathogenic because it is RpoS-defective 64, 65. RpoS is frequently lost and re-acquired in various bacteria 66, and those without it evolve control of its many functions by alternative mechanisms 67.

DSB-dependent mutation was found first in *E. coli* 51, 68 then described in bakers yeast 69–72. In yeast the mutagenicity of DSB repair is not known to be stress-inducible, and it may be constitutive. However, DSB repair itself appears to be stress inducible in the pathogenic yeast *Candida* 73, 74, making it possible that yeasts also have stress-inducible mutagenesis caused by error-prone DSB repair, but with the stress-inducibility controlled at the step of DSE creation or repair rather than the mutagenicity of repair as in *E. coli* 74.

Other stress-inducible mutation mechanisms that also require RpoS include transposition/excision of phage Mu 75, 76, stress-inducible point mutation 77, and transposition 78 in *Pseudomonas putida*, DSB-independent stress-induced mutation in aging colonies of an *E. coli* natural isolate 79, and DSB-dependent 12, 33 stress-induced gene amplification in *E. coli* 28. RpoS is induced by many different stressors including starvation, osmotic-, pH-, temperature-, and oxidative stresses 29. The importance of coupling mutation pathways to a broad general stress response like RpoS might be that genetic diversity may be generated responsively to many different stressors and environments.

## Other stress responses promote mutation

Stress-inducible mutation seems to have arisen independently many times, and appears to be a collection of different molecular mechanisms observable in various organisms (reviewed in 2). One common theme however appears to be regulation by stress responses (reviewed in 2). Bacterial starvation and other stress responses other than RpoS also promote mutagenesis during stress. These include the stringent and the competence starvation–stress responses in *Bacillus subtilis* 80, and the stringent 81, 82 and cyclic AMP 76, 83 responses to starvation, and the RpoE membrane-protein stress response 84 in *E. coli*. These promote base-substitutions 82, 83, frameshift mutations 84, amplification 84, mobile-intron movement 81, and transposon excision 76, 81. A summary of the effects of various stress responses on mutagenesis is provided in 2.

Some yeast and mammalian stress-induced mutation pathways, which respond to hypoxic-stress and heat-shock responses, are reviewed elsewhere 2–4, 73, 85, 86. All of these promote genetic diversity, and potentially the ability of cells to evolve, when stressed; futhermore, they may be important to tumor progression in hypoxic environments, resistance to stress-inducing cancer chemotherapies, and chemotherapies against pathogens. These examples illustrate the apparently multiple evolutions of mechanisms that couple genomic instability pathways with stress responses and stress (reviewed in 2). The importance of all of these is that genetic diversity is generated preferentially when cells are maladapted to their environment – when stressed. This discovery contrasts with early ideas about constant and gradual mutation underlying evolution.

## Anti-evolvability drugs?

The power of identifying the proteins and their mechanisms of action in stress-inducible mutation pathways is that with their identities comes the potential to inhibit these pathways therapeutically. In the future, we may take anti-evolution drugs to block stress-promoted adaptation of pathogens to host-instigated stressors. Such strategies would include inhibiting stress-induced mutation pathways. These fundamentally different antibiotic or antifungal drugs would work by suppressing pathogen evolution while the immune system catches up. Such drugs could be given alone. However anti-evolution drugs might be used more powerfully as co-therapies. We suggest that all current antibiotic, antifungal and even anti-cancer drugs can be viewed as a single class: “anti-proliferative.” They either kill cells or inhibit their growth, and in doing so, many are stressors. This means that by their very nature, standard chemotherapies can be expected to induce mutations that allow pathogens or cancer cells to out-evolve them. This has now been shown for antibiotics. Antibiotics of various kinds are stressors 87, 88 that induce mutations 58, 89, 90, including those conferring resistance to the same 58 or different 90 antibiotics. Anti-evolvability drugs might be powerful co-therapies that could block the mutagenic effects of standard anti-proliferative drugs, to let them work without promoting resistance 2, 58, 59. Similarly, we suggest that cancer chemotherapies that prevent hypoxia-induced- 3 and possibly traditional chemotherapy-stress-induced mutagenesis could both inhibit stress-induced mutations that fuel tumor progression and also inhibit mutation to resistance while a traditional chemotherapeutic agent does its job.

## Conclusion

Stress-induced mutation in *E. coli* has come of age. Recent work overcomes old concerns about its relevance and generality, and even suggests possible harmony between previously discordant views.

Double-strand-break-dependent stress-induced mutation in *E. coli* illustrates the potential importance of the path from molecular mechanisms to mechanisms of evolution. Basic assumptions may require revision. The molecular mechanisms of genetic and non-genetic inheritance include many yet to be dissected, but are fundamental to detailed and specific understanding of evolution, which in turn may suggest realistic strategies against evolution-driven problems such as cancer and infectious disease.

## Abbreviations

DSBAR: double-strand break
DSE: double-strand end
HR: homologous recombination
